# Colorectal cancer molecular classification using BRAF, KRAS, microsatellite instability and CIMP status: Prognostic implications and response to chemotherapy

**DOI:** 10.1371/journal.pone.0203051

**Published:** 2018-09-06

**Authors:** Oscar Murcia, Míriam Juárez, María Rodríguez-Soler, Eva Hernández-Illán, Mar Giner-Calabuig, Miren Alustiza, Cecilia Egoavil, Adela Castillejo, Cristina Alenda, Víctor Barberá, Carolina Mangas-Sanjuan, Ana Yuste, Luís Bujanda, Joan Clofent, Montserrat Andreu, Antoni Castells, Xavier Llor, Pedro Zapater, Rodrigo Jover

**Affiliations:** 1 Servicio de Medicina Digestiva, Hospital General Universitario de Alicante, Instituto de Investigación Sanitaria ISABIAL, Alicante, Spain; 2 Unidad de Investigación, Hospital General Universitario de Alicante, Instituto de Investigación Sanitaria ISABIAL, Alicante, Spain; 3 Molecular Genetics Laboratory, Hospital General Universitario de Elche, Instituto de Investigación Sanitaria ISABIAL, Alicante, Spain; 4 Department of Pathology, Hospital General Universitario de Alicante, Instituto de Investigación Sanitaria ISABIAL, Alicante, Spain; 5 Oncology Department, Hospital General Universitario de Alicante, Instituto de Investigación Sanitaria ISABIAL, Alicante, Spain; 6 Gastroenterology Unity, Hospital Donostia/Instituto Biodonostia, Centro de Investigación Biomédica en Red de Enfermedades Hepáticas y Digestivas (CIBERehd), Universidad del País Vasco (UPV/EHU), San Sebastián, Spain; 7 Gastroentyerology Unit, Hospital de Sagunto, Sagunto, Spain; 8 Gastroenterology Unit, IMIM: Institut Hospital del Mar d'Investigacions Mèdiques, Hospital del Mar, Barcelona, Spain; 9 Gastroenterology Unit, Hospital Clínic, IDIBAPS, CIBERehd, University of Barcelona, Barcelona, Spain; 10 Section of Digestive Diseases, Yale University, Yale New Haven Hospital, New Haven, Connecticut, United States of America; 11 Clinical Pharmacology Department, Hospital General Universitario de Alicante, Instituto de Investigación Sanitaria ISABIAL, Alicante, Spain; Sapporo Ika Daigaku, JAPAN

## Abstract

**Objective:**

The aim of this study was to validate a molecular classification of colorectal cancer (CRC) based on microsatellite instability (MSI), CpG island methylator phenotype (CIMP) status, *BRAF*, and *KRAS* and investigate each subtype’s response to chemotherapy.

**Design:**

This retrospective observational study included a population-based cohort of 878 CRC patients. We classified tumours into five different subtypes based on *BRAF* and *KRAS* mutation, CIMP status, and MSI. Patients with advanced stage II (T4N0M0) and stage III tumours received 5-fluoruracil (5-FU)-based chemotherapy or no adjuvant treatment based on clinical criteria. The main outcome was disease-free survival (DFS).

**Results:**

Patients with the combination of microsatellite stable (MSS) tumours, *BRAF* mutation and CIMP positive exhibited the worst prognosis in univariate (log rank P<0.0001) and multivariate analyses (hazard ratio 1.75, 95% CI 1.05–2.93, P = 0.03) after adjusting for age, sex, chemotherapy, and TNM stage. Treatment with 5-FU-based regimens improved prognosis in patients with the combination of MSS tumours, *KRAS* mutation and CIMP negative (log rank P = 0.003) as well as in patients with MSS tumours plus *BRAF* and *KRAS* wild-type and CIMP negative (log-rank P<0.001). After adjusting for age, sex, and TNM stage in the multivariate analysis, only patients with the latter molecular combination had independently improved prognosis after adjuvant chemotherapy (hazard ratio 2.06, 95% CI 1.24–3.44, P = 0.005).

**Conclusion:**

We confirmed the prognostic value of stratifying CRC according to molecular subtypes using MSI, CIMP status, and somatic *KRAS* and *BRAF* mutation. Patients with traditional chromosomally unstable tumours obtained the best benefit from adjuvant 5-FU-based chemotherapy.

## Introduction

Colorectal cancer (CRC) is one of the most prevalent neoplasms and an important cause of death in Western countries [1]. In the last few years, several therapeutic options have improved the survival of CRC patients, due in part to better comprehension of its molecular biology. CRC has largely been considered a heterogeneous disease, with an increasing number of pathways involved in different subtypes. Therefore, it is plausible that these different subtypes exhibit different behaviour in terms of not only overall prognosis, but also the response to adjuvant chemotherapy. Recently, studies demonstrating the role of immunotherapy in MSI tumours highlighted the importance of adequately classifying CRC patients in order to effectively provide the best molecular-based treatment [2].

Phipps et al [3] and Sinicrope et al [4] proposed two molecular classifications with prognostic implications based on different combinations of microsatellite instability (MSI), CpG island methylator phenotype (CIMP) status, and *BRAF* and *KRAS* somatic mutations in tumours. Thus, we can classify CRC into five subtypes according to the described pathways of colorectal carcinogenesis [5] Studies have shown clinical and pathological differences between the subtypes, as well as relevant differences in prognosis [3–7]. On the other hand, Guinney and colleagues proposed a new molecular classification based on gene expression profiling,[8;9] but some difficulties have arisen in transferring this classification to clinical practice [10–12]. As the molecular classification based on MSI, CIMP, *BRAF*, and *KRAS* [3] is simple and easy to apply, the aim of our study was to reproduce and validate this classification and investigate the response of each of subtypes to chemotherapy.

## Material and methods

### Study population

**Fig 1:**
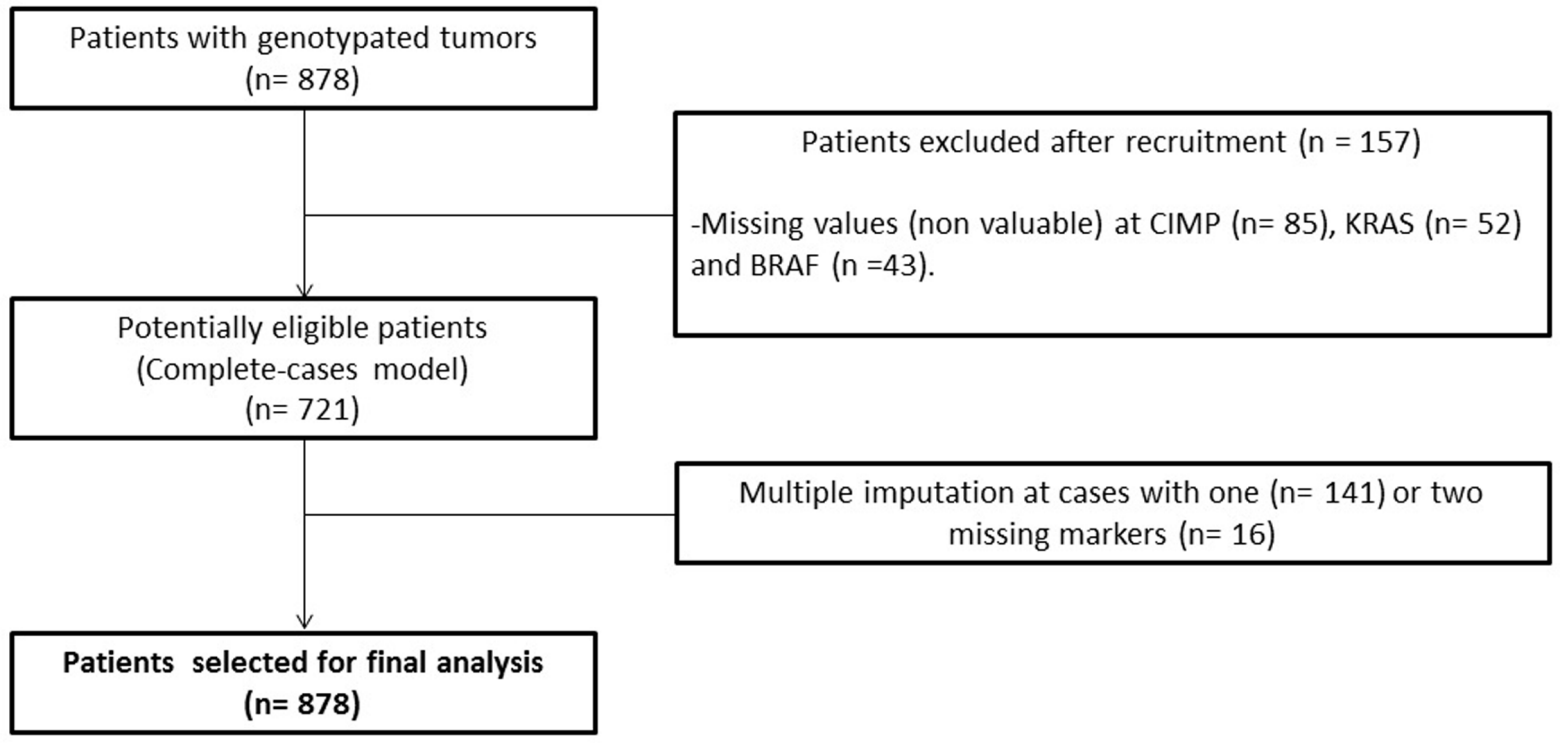
Flow diagram of patients included in the study.

We enrolled a population-based cohort of 878 patients with CRC, available tumour tissue and complete genotyping for *BRAF*, *KRAS*, CIMP and MSI status, from the nationwide and multicentre EPICOLON I and EPICOLON II projects [13;14] in a retrospective observational study (**Fig 1**). Patients were included between years 2000–2001 in EPICOLON I and 2006–2007 in EPICOLON II. Adjuvant chemotherapy was administered following standard clinical criteria, regardless the MMR status of the tumours. Oncologists that decided treatment with adjuvant chemotherapy were blinded to the MMR status of the tumours. The institutional review board (IRB) of the Hospital General Universitario de Alicante approved the study as well as IRBs of all the participant hospitals. All patients provided written informed consent.

### CRC samples

After the surgery of each CRC, tumor samples were obtained and paraffin-embedded at the different participant hospitals. These paraffin-embedded samples were sent to the Hospital General Universitario of Alicante. There, DNA was extracted from paraffin-embedded tissue with the QIAamp DNA Investigator kit (QIAGEN, Hilden, Germany) and with the E.Z.N.A Forensic DNA kit (OMEGA Biotek, Norcross, GA), according to manufacturer’s protocols.

### Variables

We analysed patient age, sex, tumour location, date and stage at the time of CRC diagnosis, *BRAF* and *KRAS* mutation status, CIMP, and MSI status, treatment (surgical, chemotherapy, radiotherapy, or biologic), and disease-free survival (DFS) time in months. We analysed the response to adjuvant chemotherapy in patients with advanced stage II (TNM stage II and T4) and stage III tumours. The main outcome was DFS. The follow-up of patients after surgery was performed according to clinical practice guidelines with scheduled CEA measurements, computed tomography and colonoscopy procedures [15;16].

### Mutations in the BRAF and KRAS genes

The V600E *BRAF* mutation was detected by real-time PCR (ABI PRISM 7500, Applied Biosystems, Foster City, CA) using specific TaqMan probes and allelic discrimination software as described previously [17]. The reporter fluorophore on the probes was 6-carboxyfluorescein (FAM) for the mutant allele and VIC for the wild-type allele. We used the allelic discrimination software on the ABI Prism 7500 instrument to analyse the fluorescence data. *KRAS* mutation at exon 1, including codons 12 and 13, was identified by DNA direct sequencing. We assessed both mutations by direct amplicon sequencing with BigDye v1.1 terminators and a 3500 Genetic Analyzer (Applied Biosystems) [18].

### CIMP analysis

Genomic DNA was modified with sodium-bisulphite using the EZ Methylation Gold Kit (Zymo Research, Orange, CA). The PCR reaction contained bisulphite modified DNA, HotStar Taq polymerase, forward primers, biotinylated reverse primers, and water. We analysed markers by real-time methylight technology, considering a tumour CIMP-H when three of the five analysed markers (*CACNAG1*, *SOCS1*, *RUNX3*, *NEUROG1 and MLH1*) harboured aberrant hypermethylation. Each marker was classified as methylated when the mean percentage was greater than 5% for *CACNAG1*, *SOCS1*, *RUNX3*, and *MLH1*, and 10% for *NEUROG1*.

### MSI analysis

We tested for MSI using either the 5-marker panel proposed by the National Cancer Institute or a pentaplex of mononucleotide repeats (BAT25, BAT26, NR21, NR24, and NR27), classifying tumours as microsatellite stable (MSS) or unstable (MSI). The presence of two or more unstable markers defined a tumour as MSI [19;20].

### Chemotherapy regimen

The adjuvant chemotherapy regimen followed standard clinical criteria. Patients with advanced stage II and stage III tumours received 5-fluoruracil (5-FU) based chemotherapy (5-FU, capecitabine, 5-FU plus oxaliplatin (FOLFOX), or 5-FU plus irinotecan (FOLFIRI)) as first line treatment following standard schedules and doses according to the local decision of the responsible oncologist. Oncologists were blinded to the *BRAF*, *KRAS*, MSI or CIMP tumour status. The decision to give or not to give chemotherapy drugs was based on the oncologist criteria in each of the participating centres.

### Classification of CRC

We classified tumours into five subtypes according to the model proposed by Phipps et al [3] and shown in **Table 1**. Subtype 1, sporadic serrated and unstable tumours; subtype 2, sporadic serrated tumours without MSI; subtype 3, traditional tumours with *KRAS* mutations or serrated tumours from the alternative serrated pathway; subtype 4, traditional chromosomally unstable (CIN) tumours without *KRAS* mutations; and subtype 5, familial unstable tumours.

**Table 1:** Molecular classification of colorectal cancer. MSI, microsatellite instability; MSS, microsatellite stability.

	MMR	CIMP	BRAF	KRAS
Subtype 1	MSI	+	+	-
Subtype 2	MSS	+	+	-
Subtype 3	MSS	-	-	+
Subtype 4	MSS	-	-	-
Subtype 5	MSI	-	-	-

### Statistical analysis

In order to maximize the number of cases available for analysis, we performed a multiple imputation model to complete cases with missing values at one (n = 141) or two molecular markers (n = 16) [3;21]. We carried out 25 rounds of imputation using the automatic model of SPSS software until an appropriate model was obtained [22]. We considered a deviation proportion with respect to original data <0.1% as appropriate; it reliably reproduced the percentage of alterations at the four markers in the complete cases model. The imputation took into account *BRAF* and *KRAS* status, presence of CIMP, MMR status, sex, age, TNM stage, tumour location, treatment with chemotherapy, and DFS time. After imputation, we classified cases into subtypes 1 to 5. Cases with no defined molecular combination were defined as the “unclassified” subtype. We performed several simulation analyses before concluding the validation of the imputation model, including relative risks (RRs) and hazard ratios (HRs). Original data, non-including patients with missing values using the imputation model, are available as Supplementary material and referred as complete-cases model (**S1–S4 Tables**).

Parametric continuous variables are reported as mean ± standard deviation (SD) and nonparametric continuous variables as median (Q2-Q3 interquartile range). We compared categorical variables using chi-squared.

For overall prognosis, we compared differences in DFS time (interval of time between remission of disease and their reappearance) among the five subtypes by log rank test in a univariate analysis, expressing it graphically with Kaplan-Meier survival curves. The multivariate analysis was performed by adjusting for potential confounder and interaction variables (age, sex, TNM stage, and chemotherapy) in a Cox regression model. Subtype 4 was the subtype of reference.

For the adjuvant chemotherapy response, we performed several univariate analyses for each subtype. The multivariate analysis was adjusted for the different subtypes, age, sex and TNM stage. As comparisons were made in each subgroup between patients receiving and not receiving adjuvant chemotherapy, we did not establish a reference group.

All P-values were two-sided, with P<0.05 considered significant. All data were analysed using SPSS 22.0 software.

## Results

Among the 878 patients, complete analysis of the four molecular markers was possible in 721 (**Fig 1**). A total of 141 cases had one missed marker, and 16 cases had two missed markers. These cases were completed using the multiple imputation model. The median follow-up was 52 months (interquartile range 16–64). A total of 241 cases (27%) had a CIMP-high pattern. A total of 324 cases had a somatic *KRAS* mutation (37%) and 54 had somatic mutations in *BRAF* (6%). Eighty cases (10%) had MSI. According to the proposed model, we could classify 677 patients (77%) into subtypes 1 to 5: 25 (3%) subtype 1, 22 (3%) subtype 2, 218 (25%) subtype 3, 388 (44%) subtype 4, and 24 (3%) subtype 5. The remaining cases (n = 201, 23%) were tumours of the “unclassified” molecular subtype (**Table 2**). The median age was lower in cases with subtype 5 tumours compared to the other subtype groups, which had similar median ages. Sex was homogenously distributed, except in the subtype 5 group, which had a predominance of women. The majority of tumours were TNM stages II or III at diagnosis, with no significant differences among the subtypes. A majority of subtype 1 tumours were right-sided, whereas subtypes 3 and 4 were preferably located in the left colon. The proportion of patients treated with chemotherapy differed slightly among subtypes (**Table 2**).

**Table 2:** Clinical characteristics of patients according to subtype in multiple imputation model. St, subtype. DFS, disease-free survival.

		St 1 (n = 25 [2.8%])	St 2 (n = 22 [2.5%])	St 3 (n = 218 [24.8%])	St 4 (n = 388 [44.2%])	St 5 (n = 24 [2.7%])	Unclassified (n = 201 [22.9%])
Median of age (years)		77	74	73	72	66	73
**Age at diagnosis (years, %)**	<40	0 (0)	0 (0)	2 (0.9)	2 (0.5)	0 (0)	2 (1.0)
	40–49	0 (0)	1 (4.5)	14 (6.4)	11 (2.8)	3 (12.5)	4 (2.0)
	50–59	2 (8.0)	2 (9.1)	17 (7.8)	41 (10.6)	5 (20.8)	17 (8.5)
	60–69	9 (36.0)	4 (18.2)	39 (17.9)	99 (25.5)	6 (25.0)	43 (19.7)
	>70	14 (56.0)	15 (68.2)	146 (67.0)	235 (60.6)	10 (41.7)	136 (67.2)
**Sex, n (%)**	Male	12 (48.0)	10 (50.0)	134 (61.5)	245 (63.1)	8 (33.3)	111 (55.2)
	Female	13 (52.0)	10 (50.0)	84 (38.5)	143 (36.9)	16 (66.7)	90 (44.8)
**TNM stage at diagnosis**	I	3 (12.0)	1 (4.5)	45 (20.6)	53 (13.7)	6 (25.0)	30 (14.9)
	II	14 (56.0)	3 (13.6)	68 (31.2)	157 (40.5)	13 (54.2)	71 (35.3)
	III	7 (28.0)	10 (45.5)	67 (30.7)	124 (32.0)	2 (8.3)	70 (34.8)
	IV	1 (4.0)	8 (36.4)	38 (17.4)	54 (13.9)	3 (12.5)	30 (14.9)
**Tumor location, n (%)**	Right colon	23 (92.0)	13 (59.1)	69 (31.7)	66 (17.0)	14 (58.3)	91 (45.3)
	Left colon	2 (8.0)	9 (40.9)	149 (68.3)	322 (83.0)	10 (41.7)	110 (54.7)
**1st line Chemotherapy**	5-FU or Capecitabine	5 (20.0)	9 (40.9)	53 (24.3)	116 (29.9)	9 (37.5)	39 (19.4)
	FOLFOX	3 (12.0)	3 (13.6)	34 (15.6)	88 (22.7)	2 (8.3)	45 (22.4)
	No CT	17 (68.0)	10 (45.5)	131 (60.1)	184 (47.4)	13 (54.2)	117 (58.2)
**DFST, months (median)**		60.0	18.0	38.7	50.9	62.8	54.8

Regarding the “unclassified” subtype”, there were observed three major genetic combinations among the 201 cases. Of them, 93 (46.3%) had CIMP-H and KRAS-mutation, which were equally distributed regarding sex, age and tumor location. Other 68 cases (33.8%) had CIMP-H in combination with any other genetic alteration. These were preferentially men (63%) and located in left colon (71%). Finally, 21 showed CIMP-H and MSI combination with BRAF wild-type (10.4%). Characteristics of the “unclassified” subtype are shown in the **S1 Table.**

### Overall prognosis

**Fig 2:**
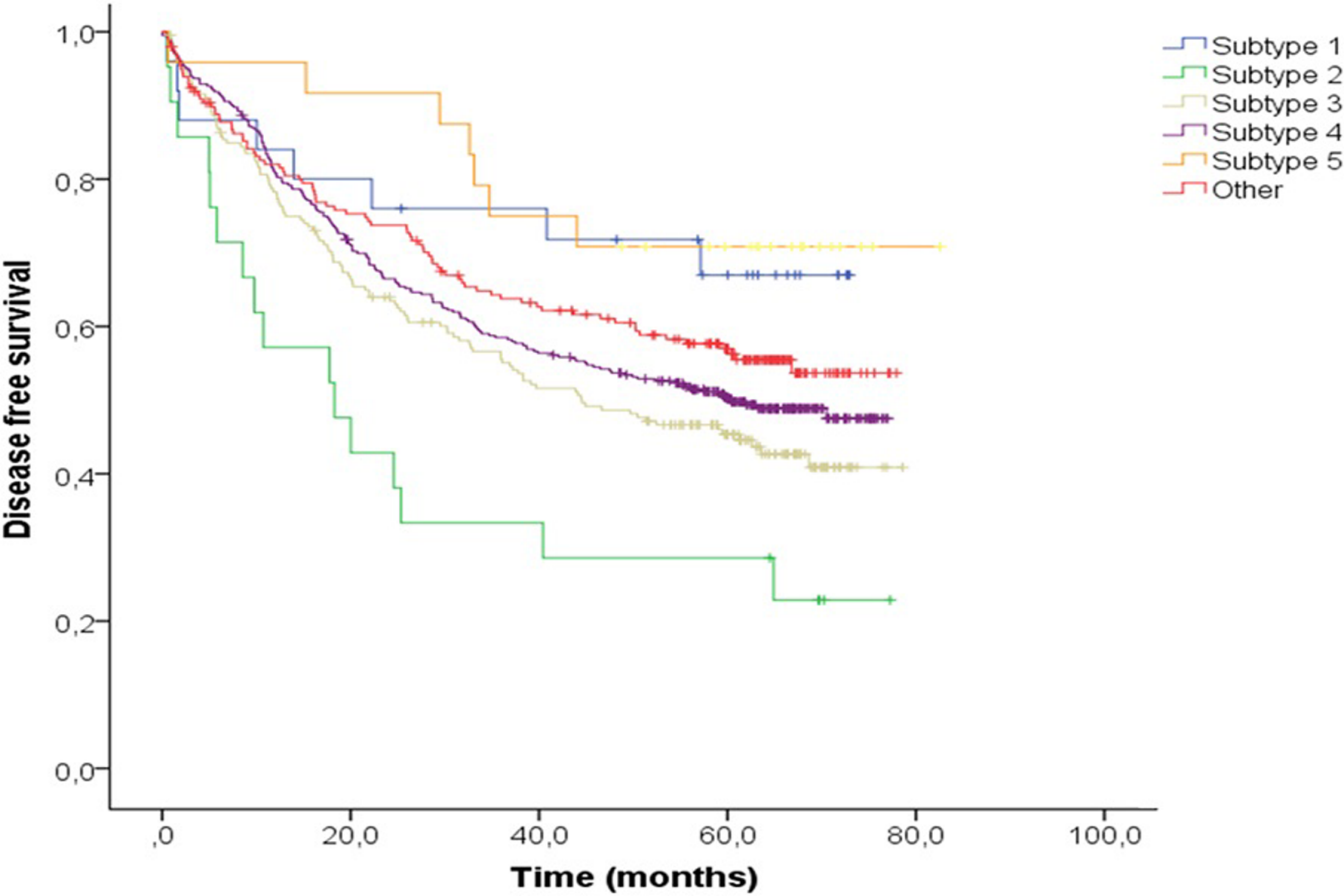
Kaplan-Meier survival curves comparing disease-free survival in colorectal cancer patients by tumour subtype.

**Fig 2** shows the Kaplan Meier survival curves for the different CRC subtypes. In the univariate analysis, subtype 2 had the lowest DFS, below 30%. In contrast, subtype 5 had a DFS rate at the end of the follow-up period of up to 80% (log rank P<0.001, **Table 3**).

**Table 3:** Overall prognosis for different subtypes in the multiple imputation model. Subtype 4 serves as a reference. The analysis was adjusted for sex, age, chemotherapy, and TNM as potential confounder factors. CRC, colorectal cancer; HR, hazard ratio; CI, confidence interval.

	Case participants		Relapse or CRC-death				
	Number	%	Number	%	HR	95% CI	P value
**Subtype 1**	25	2.8	8	32.0	0.59	0.29–1.20	0.144
**Subtype 2**	22	2.5	16	76.2	1.75	1.05–2.93	0.032
**Subtype 3**	218	24.8	117	54.9	1.21	0.96–1.52	0.109
**Subtype 4**	388	44.2	191	50.3	1.0	Ref	—
**Subtype 5**	24	2.7	7	29.2	0.56	0.26–1.19	0.131
**Unclassified**	201	22.9	85	42.9	0.84	0.65–1.09	0.187

Multivariate analysis showed that age (P<0.001), TNM stage (P<0.001), and chemotherapy (P<0.001) were significantly influential in regards to DFS. Regarding the different subtypes, only patients with subtype 2 (HR = 1.75, P = 0.03) had a different prognosis with respect to the subtype of reference after adjusting for age, sex, chemotherapy, and TNM (**Table 3**). Subtype 3 also revealed a trend for different prognosis (HR = 1.2, P = 0.109). Trends were similar in the complete-cases model, although no significantly independent better prognosis was found in subtype 2 (**S2 Table**).

### Prognosis after adjuvant chemotherapy

We analysed the response to chemotherapy in 324 patients with advanced stage II (T4N0M0) or stage III CRC (**Table 4**).

**Table 4:** Clinical characteristics of patients with advanced stage II and stage III tumours in multiple imputation model. DFST, disease-free survival time. St, Subtype.

		St 1 (n = 9 [2.8%])	St 2 (n = 10 [3.1%])	St 3 (n = 77 [23.8%])	St 4 (n = 142 [43.8%])	St 5 (n = 6 [1.9%])	Unclassified (n = 80 [24.7%])
**Median of age (years)**		74	74	72	72	72	73
**Age at diagnosis (years, %)**	<40	0 (0)	0 (0)	0 (0)	0 (0)	0 (0)	1 (1.3)
	40–49	0 (0)	0 (0)	4 (5.2)	3 (2.1)	1 (16.7)	1 (1.3)
	50–59	0 (0)	1 (10.0)	8 (10.4)	15 (10.6)	0 (0)	4 (5.0)
	60–69	4 (44.4)	2 (20.0)	16 (20.8)	32 (22.5)	2 (33.3)	18 (22.5)
	>70	5 (55.6)	7 (70.0)	49 (65.6)	92 (64.8)	3 (50.0)	56 (70.0)
**Sex, n (%)**	Male	4 (44.4)	4 (40.0)	40 (51.9)	85 (59.9)	2 (33.3)	36 (45.0)
	Female	5 (55.6)	6 (60.0)	37 (48.1)	57 (40.1)	4 (66.7)	44 (55.0)
**TNM stage at diagnosis**	II	2 (22.2)	0 (0)	10 (13.0)	18 (12.7)	4 (66.7)	10 (12.5)
	III	7 (77.8)	10 (100)	67 (87.0)	124 (87.3)	2 (33.3)	70 (87.5)
**Tumor location, n (%)**	Right colon	7 (77.8)	6 (60.0)	24 (31.2)	25 (17.6)	4 (66.7)	35 (43.8)
	Left colon	2 (22.2)	4 (40.0)	53 (68.8)	117 (82.4)	2 (33.3)	45 (56.2)
**1st line Chemotherapy**	5-FU or Capecitabine	3 (33.3)	4 (40.0)	25 (32.5)	50 (35.2)	3 (50.0)	22 (27.5)
	FOLFOX	1 (11.1)	2 (20.0)	22 (28.6)	49 (34.5)	0 (0)	29 (36.3)
	No CT	5 (55.5)	4 (40.0)	30 (39.0)	43 (30.3)	3 (50.0)	29 (36.3)
**DFST, months (median)**		57.1	32.5	30.9	46.7	47.8	55.7

**Fig 3:**
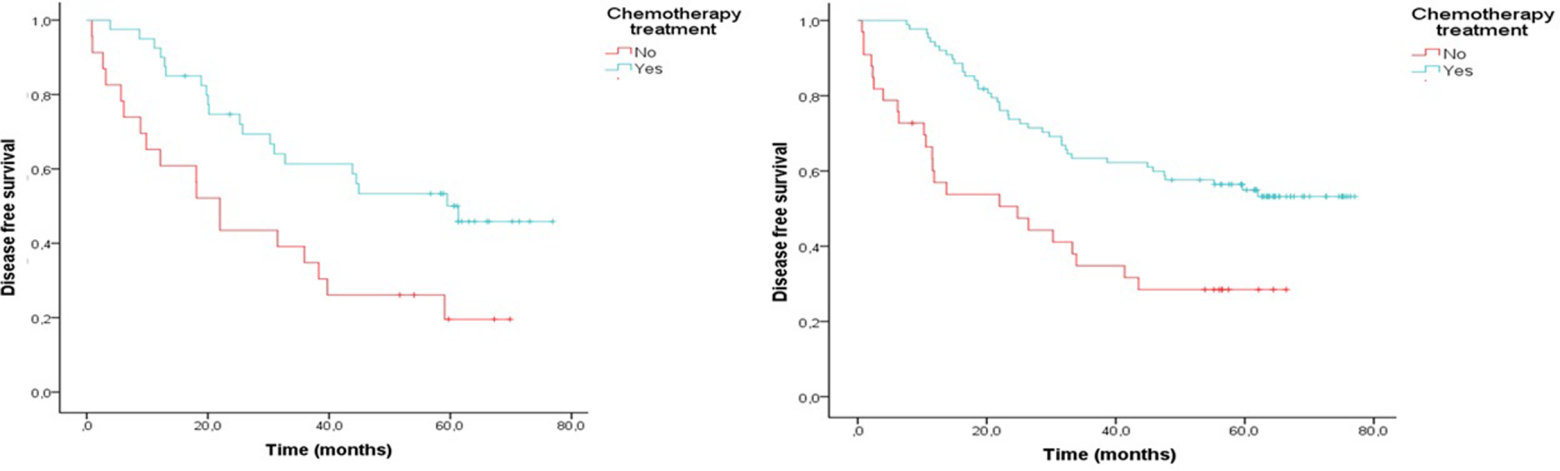
Disease-free survival in patients with advanced stage II and stage III colorectal cancer according to whether they received adjuvant chemotherapy. A) subtype 3 patients and B) subtype 4 patients.

In this group, a total of 114 patients did not receive adjuvant chemotherapy. Overall, chemotherapy improved DFS time (log rank P<0.001) in the univariate analysis. Regarding CRC subtypes, there was a clear benefit from chemotherapy in subtypes 3 and 4. In both subtypes, the patients had higher DFS rates when they received chemotherapy (log rank P = 0.003 for subtype 3 and P<0.001 for subtype 4, **Fig 3A and 3B** and **Table 5**).

**Table 5:** Chemotherapy response of different subtypes in univariate and multivariate analyses using the multiple imputation model. The multivariate analysis was adjusted for sex, age, and TNM stage for each subtype. CT, chemotherapy; DFS, disease-free survival; HR, hazard ratio; CI, confidence interval.

		Univariate analysis			Multivariate analysis		
	CT	Number of patients	Median of DFS time (months)	P value	HR	95% CI	P value
**Subtype 1**	Yes	4	39.3	0.620	0.53	0.19–1.46	0.220
	No	5	62.2				
**Subtype 2**	Yes	6	44.6	0.068	0.40	0.04–3.92	0.430
	No	4	9.6				
**Subtype 3**	Yes	47	49.5	0.003	1.93	0.86–4.34	0.111
	No	30	27.8				
**Subtype 4**	Yes	99	54.2	0.000	2.06	1.24–3.44	0.005
	No	43	29.0				
**Subtype 5**	Yes	3	63.9	0.107	1.01	0.31–3.28	0.991
	No	3	16.9				
**Unclassified**	Yes	51	58.6	0.015	1.71	0.74–3.96	0.208
	No	29	39.0				

A multivariate analysis was also performed for the different subtypes (**Table 5**). After adjusting for age, sex, and TNM stage, only patients with subtype 4 CRC independently exhibited significant benefit with chemotherapy (subtype 3: HR = 1.93, 95% CI 0.86–4.34, P = 0.1; subtype 4: HR = 2.06, 95% CI 1.24–3.44, P = 0.005). These results were similar in the complete-cases model (**S3 and S4 Tables**).

## Discussion

Our results confirm that, as suggested in previous studies [3;4], classification of the molecular profile of CRC using *BRAF*, *KRAS*, MSI, and CIMP status can identify differences in prognosis. Our results confirm the poorest prognosis found for subtype 2 in previous cohorts, unaffected by age or TNM stage. This lower survival rate for subtype 2 tumours suggests that the combination of CIMP and *BRAF* mutation confers a poor prognosis in stable tumours, whereas the same combination in tumours with MSI (i.e., subtype 1) evolves in a completely different way. Moreover, our results show that the more common subtypes 3 and 4 benefit from adjuvant 5-FU-based chemotherapy, but only in subtype 4 CRC patients is this benefit independent of sex, age, and TNM stage. The response in other subtypes cannot be evaluated due to their small representation in our cohort.

Subtypes 1 and 2 belong to the serrated pathway of carcinogenesis, with serrated polyps as the precursor lesion, whereas subtypes 4 and 5 follow the traditional adenoma-carcinoma pathway with MSS (subtype 4) tumours or MSI (subtype 5). On the other hand, subtype 3 includes tumours that can originate in serrated or adenomatous polyps, with the *KRAS* somatic mutation as their molecular hallmark [5]. Different studies have shown discordant results with respect to the prognostic value of some molecular markers [23;24]. *BRAF* V600E has traditionally been linked to a poor prognosis, with many authors proposing it as an independent predictor of low survival [25]. However, other studies have found differences according to MMR status, showing better survival rates in tumours with MSI than in MSS tumours.[26] Something similar has been seen with CIMP [27]; though many studies have not attributed any prognostic value [23], others proposed it is a predictor of short survival, and even of high survival if MSI appeared concomitantly [28]. Taken together, all these discordances could be due to the consideration of isolated markers instead of combinations of them [10]. Grouping according to the proposed molecular pathways led to different and well defined survival curves. Recently, Guinney et al [8] proposed a new molecular classification of CRC based on gene expression datasets. This classification divides CRC into four subtypes: MSI immune with MSI, *BRAF* mutated, and CIMP positive tumours; canonical with *WNT* and *MYC* activation; metabolic with *KRAS* mutated tumours; and mesenchymal, characterized by stromal infiltration and TGF-β activation. The majority of the proposed molecular subtypes somehow coincide with those in our study [10]. However, some patients are very difficult to classify, especially those from the mesenchymal subtype, which lacks convenient molecular makers. Although this classification can be considered the most robust classification system currently available for CRC, with a clear biological basis and comprehensive tumour characterization based on advances in genomic technology, the challenges in data interpretation and clinical application have not yet been adequately overcome [10].

In our knowledge, this is the first study that examines the classification of CRC proposed by Phipps and tries to correlate the response of each subtype to chemotherapy and compare among them. Previous studies using these classifications did not analyse the role of chemotherapy in the evolution of CRC patients in regards to molecular subtypes. Unfortunately, our study does not have enough power to confirm this lack of response of MSI subtypes 1 and 5 due to the small sample size. However, this lack of response to 5-FU-based chemotherapy by tumours with MSI was well established in previous studies [29–32]. Our results highlight that the more common CRC subtypes seem to improve with chemotherapy in the univariate analysis (subtypes 3 and 4). These benefits were only maintained in subtype 4 in the multivariate analysis, revealing an advantage of this subtype in 5-FU-based chemotherapy, and a potential limited effect of this treatment in subtype 3 CRC patients. On the other hand, we also observed a trend of obtaining a benefit from chemotherapy for subtype 2. The hypothesis that patients with molecularly different tumours have different responses to chemotherapy is clearly plausible given the biological diversity found between CRC tumours. It is especially interesting to investigate the response of subtype 2 and 3 CRC, as they are biologically different from the classical CIN CRC. Another point that requires specific research is the possible role of new targeted molecules in the different molecular CRC subtypes. Improved survival using immunotherapy in CRC with MSI was demonstrated recently [2], and there is a biological explanation for both: the lack of effect of conventional 5-FU-based chemotherapy and the benefit from immunotherapy in this subtype of CRC. The role of immunotherapy or other types of chemotherapy could be more appropriately investigated if we were able to adequately classify CRC in different subtypes. Larger cohorts are needed for differentially investigating the role of different drugs in the diverse molecular landscape of CRC.

This study has some limitations that should be considered. First, it is important to note that we could appropriately classify only 71% of these cases using the molecular markers. There are at least two reasons for this. First, biology is not perfect and may include aberrant combinations with an unpredictable course. Second, it is possible that there are other molecular pathways of colorectal carcinogenesis not included in this classification and molecular subtypes of CRC with prognostic implications could include more than those proposed. Another limitation of our study is the small number of patients receiving adjuvant chemotherapy in minority subtypes (i.e., 1, 2, and 5), precluding the possibility of appropriately determining the response to 5-FU-based chemotherapy in these subtypes. In addition, our study shows a higher proportion of CIMP positive tumors (27%) than other studies [3]. This could be likely attributable to the characteristics of our sample, such as the mean age of our unselected consecutive population, with more than 60% older than 70 years-old. Finally, the decision regarding giving chemotherapy in stage II and III patients was based on clinical criteria. Previous studies of these CRC subtypes were nested in randomized clinical trials comparing different chemotherapy regimens [3,4], which precludes obtaining information about the predictive value of this subtype classification. We can investigate the role of adjuvant 5-FU-based chemotherapy only if there is a group of patients that do not receive treatment, and that could be considered a strength of our study. However, basing the treatment decision on clinical criteria can provoke some biases, because it is possible that this clinical decision was based on factors such as age and performance status, avoiding chemotherapy in people in the worst health status. Nevertheless, that scenario otherwise allows us to study the effect of adjuvant chemotherapy in the different subtypes considering the difficulty obtaining other data and given that it could be considered unethical to design a randomized clinical trial with an untreated control group in advanced stage II and stage III CRC patients.

In summary, our results confirm the prognostic value of stratifying CRC according to molecular subtypes using MSI and CIMP status and somatic *KRAS* and *BRAF* mutations. The use of this classification can be helpful when making clinical decisions in the management of CRC patients. Serrated CRC with CIMP and somatic *BRAF* mutation has the worst prognosis. 5-FU-based chemotherapy provides a benefit in subtypes 3 and 4 in regards to DFS time, but this benefit was independent after adjusting for sex, age, and TNM stage only in subtype 4 tumours representing the traditional CIN pathway. A better understanding of the biology of CRC, description of new pathways of colorectal carcinogenesis, and determination of driver mutations will improve and complete this classification in the near future. Research should focus on molecularly targeted therapy for the different subtypes, especially for those with the worst prognosis.

## Supporting information

S1 Table Clinical characteristics of patients according to subtype in complete-cases model. St, subtype. DFS, disease-free survival.
(DOCX)

S2 Table Overall prognosis for different subtypes in the complete cases model. Subtype 4 serves as a reference. The analysis was adjusted for sex, age, chemotherapy, and TNM as potential confounder factors. CRC, colorectal cancer; HR, hazard ratio; CI, confidence interval.
(DOCX)

S3 Table Clinical characteristics of patients with advanced stage II and stage III tumours in complete-cases model. DFST, disease-free survival time. St, Subtype.
(DOCX)

S4 Table Chemotherapy response of different subtypes in univariate and multivariate analyses using the complete-cases model. The multivariate analysis was adjusted for sex, age, and TNM stage for each subtype. CT, chemotherapy; DFS, disease-free survival; HR, hazard ratio; CI, confidence interval.
(DOCX)
