# Supplementary material for: Colorectal cancer molecular classification using BRAF, KRAS, microsatellite instability and CIMP status: Prognostic implications and response to chemotherapy
Source: PLoS One. 2018 Sep 6;13(9):e0203051. doi: 10.1371/journal.pone.0203051 (PMC6126803; doi:10.1371/journal.pone.0203051)
Supplement: S1 Table — St, subtype. DFS, disease-free survival. (DOCX) [file pone.0203051.s001.docx]

**Supplementary Table 1. Clinical characteristics of patients according to subtype in complete-cases model.** St, subtype. DFS, disease-free survival.

|  |  | **St 1**  **(n=20 [2.8%])** | **St 2**  **(n= 16 [2.2%])** | **St 3**  **(n= 188 [26.1%])** | **St 4**  **(n= 315 [43.7%])** | **St 5**  **(n= 18 [2.5%])** | **Unclassified (n= 164 [22.7%])** |
| --- | --- | --- | --- | --- | --- | --- | --- |
| **Median of age (years)** |  | 76 | 75 | 73 | 72 | 65 | 72 |
| **Age at diagnosis (years, %)** | <40  40-49  50-59  60-69  >70 | 0 (0)  0 (0)  2 (10.0)  7 (35.0)  11 (55.0) | 0 (0)  1 (6.3)  2 (12.5)  3 (18.8)  10 (62.5) | 1 (0.5)  12 (6.4)  16 (8.5)  32 (17.0)  127 (67.6) | 2 (0.6)  5 (1.6)  31 (9.8)  74 (23.5)  203 (64.4) | 0 (0)  3 (16.6)  3 (16.6)  6 (33.3)  9 (50.0) | 2 (1.2)  4 (2.4)  10 (6.1)  33 (20.1)  115 (70.1) |
| **Sex, n (%)** | Male  Female | 10 (50.0)  10 (50.0) | 9 (56.3)  7 (43.8) | 121 (64.4)  67 (35.6) | 199 (63.2)  116 (36.8) | 7 (38.9)  11 (61.1) | 89 (54.3)  75 (45.7) |
| **TNM stage at diagnosis** | I  II  III  IV | 2 (10.0)  12 (60.0)  5 (25.0)  1 (5.0) | 1 (6.3)  3 (18.8)  6 (37.5)  6 (37.5) | 40 (21.3)  57 (30.3)  56 (29.8)  35 (18.6) | 43 (13.7)  128 (40.6)  106 (33.7)  38 (12.1) | 4 (22.2)  10 (55.6)  1 (5.6)  3 (16.7) | 27 (16.5)  61 (37.2)  55 (33.5)  21 (12.8) |
| **Tumor location, n (%)** | Right colon  Left colon | 18 (90.0)  2 (10.0) | 10 (62.5)  6 (37.5) | 61 (32.4)  127 (67.6) | 57 (18.1)  258 (81.9) | 10 (55.6)  8 (44.4) | 78 (47.6)  86 (52.4) |
| **1st line Chemotherapy** | 5-FU or Capecitabine  FOLFOX  No CT | 1 (5.0)  3 (15.0)  16 (80.0) | 2 (12.5)  2 (12.5)  12 (75.0) | 14 (7.4)  28 (14.9)  146 (77.7) | 54 (17.1)  70 (22.2)  191 (60.6) | 5 (27.8)  2 (11.1)  11 (61.1) | 12 (7.3)  36 (22.0)  116 (70.7) |
| **DFST, months (median)** |  | 61.1 | 18.9 | 38.3 | 50.4 | 63.9 | 58.1 |
