# Supplementary material for: Colorectal cancer molecular classification using BRAF, KRAS, microsatellite instability and CIMP status: Prognostic implications and response to chemotherapy
Source: PLoS One. 2018 Sep 6;13(9):e0203051. doi: 10.1371/journal.pone.0203051 (PMC6126803; doi:10.1371/journal.pone.0203051)
Supplement: S2 Table — Subtype 4 serves as a reference. The analysis was adjusted for sex, age, chemotherapy, and TNM as potential confounder factors. CRC, colorectal cancer; HR, hazard ratio; CI, confidence interval. (DOCX) [file pone.0203051.s002.docx]

**Supplementary Table 2.**  **Overall prognosis for different subtypes in the complete-cases model.** Subtype 4 serves as a reference. The analysis was adjusted for sex, age, chemotherapy, and TNM as potential confounder factors. CRC, colorectal cancer; HR, hazard ratio; CI, confidence interval.

|  | **Case participants** | | **Relapse or CRC-death** | | | | |
| --- | --- | --- | --- | --- | --- | --- | --- |
|  | **Number** | **%** | **Number** | **%** | **HR** | **95% CI** | **P value** |
| **Subtype 1** | 20 | 2.8 | 6 | 30.0 | 0.57 | 0.25-1.29 | 0.175 |
| **Subtype 2** | 16 | 2.2 | 10 | 66.7 | 1.77 | 0.93-3.36 | 0.082 |
| **Subtype 3** | 188 | 26.1 | 103 | 56.0 | 1.25 | 0.97-1.60 | 0.085 |
| **Subtype 4** | 315 | 43.7 | 156 | 50.3 | 1.0 | Ref | -- |
| **Subtype 5** | 18 | 2.5 | 4 | 22.2 | 0.45 | 0.17-1.21 | 0.113 |
| **Unclassified** | 164 | 22.7 | 64 | 39.8 | 0.79 | 0.59-1.07 | 0.127 |
