# Supplementary material for: Colorectal cancer molecular classification using BRAF, KRAS, microsatellite instability and CIMP status: Prognostic implications and response to chemotherapy
Source: PLoS One. 2018 Sep 6;13(9):e0203051. doi: 10.1371/journal.pone.0203051 (PMC6126803; doi:10.1371/journal.pone.0203051)
Supplement: S3 Table — DFST, disease-free survival time. St, Subtype. (DOCX) [file pone.0203051.s003.docx]

**Supplementary Table 3. Clinical characteristics of patients with advanced stage II and stage III tumours in complete-cases model.** DFST, disease-free survival time. St, Subtype.

|  |  | **St 1**  **(n=7 [2.6%])** | **St 2**  **(n= 16 [2.2%])** | **St 3**  **(n= 65 [24.3%])** | **St 4**  **(n= 121 [45.3%])** | **St 5**  **(n= 3 [1.1%])** | **Unclassified (n= 65 [24.3%])** |
| --- | --- | --- | --- | --- | --- | --- | --- |
| **Median of age (years)** |  | 74 | 74 | 72 | 72 | 63 | 72 |
| **Age at diagnosis (years, %)** | <40  40-49  50-59  60-69  >70 | 0 (0)  0 (0)  0 (0)  3 (42.9)  4 (57.1) | 0 (0)  0 (0)  1 (16.7)  1 (16.7)  4 (66.7) | 0 (0)  4 (6.2)  7 (10.8)  11 (16.9)  43 (66.2) | 0 (0)  3 (2.5)  15 (12.4)  26 (21.5)  77 (63.6) | 0 (0)  1 (33.3)  0 (0)  2 (66.7)  0 (0) | 1 (1.5)  1 (1.5)  4 (6.2)  14 (21.5)  45 (69.2) |
| **Sex, n (%)** | Male  Female | 4 (57.1)  3 (42.9) | 3 (50.0)  3 (50.0) | 35 (53.8)  30 (46.2) | 75 (62.0)  46 (38.0) | 2 (66.7)  1 (33.3) | 31 (47.7)  34 (52.3) |
| **TNM stage at diagnosis** | II  III | 2 (28.6)  5 (71.4) | 0 (0)  6 (100.0) | 9 (13.8)  56 (86.2) | 15 (12.4)  106 (87.6) | 2 (66.7)  1 (33.3) | 10 (15.4)  55 (84.6) |
| **Tumor location, n (%)** | Right colon  Left colon | 5 (71.4)  2 (28.6) | 4 (66.6)  2 (33.3) | 23 (35.4)  42 (64.6) | 23 (19.0)  98 (81.0) | 2 (66.7)  1 (33.3) | 30 (46.2)  35 (53.8) |
| **1st line Chemotherapy** | 5-FU or Capecitabine  FOLFOX  No CT | 3 (42.9)  1 (14.3)  3 (42.9) | 3 (50.0)  1 (16.7)  2 (33.3) | 21 (32.3)  19 (29.2)  25 (38.4) | 48 (39.7)  40 (33.1)  33 (27.3) | 3 (100.0)  0 (0)  0 (0) | 21 (32.3)  24 (36.9)  20 (30.8) |
| **DFST, months (median)** |  | 56.8 | 44.5 | 32.7 | 47.5 | 71.9 | 55.6 |
