# Supplementary material for: Colorectal cancer molecular classification using BRAF, KRAS, microsatellite instability and CIMP status: Prognostic implications and response to chemotherapy
Source: PLoS One. 2018 Sep 6;13(9):e0203051. doi: 10.1371/journal.pone.0203051 (PMC6126803; doi:10.1371/journal.pone.0203051)
Supplement: S4 Table — The multivariate analysis was adjusted for sex, age, and TNM stage for each subtype. CT, chemotherapy; DFS, disease-free survival; HR, hazard ratio; CI, confidence interval. (DOCX) [file pone.0203051.s004.docx]

**Supplementary Table 4. Chemotherapy response of different subtypes in univariate and multivariate analyses using the complete-cases model.** The multivariate analysis was adjusted for sex, age, and TNM stage for each subtype. CT, chemotherapy; DFS, disease-free survival; HR, hazard ratio; CI, confidence interval.

|  | **Univariate analysis** | | | | **Multivariate analysis** | | |
| --- | --- | --- | --- | --- | --- | --- | --- |
|  | **CT** | **Number of patients** | **Median of DFS time (months)** | **P value** | **HR** | **95% CI** | **P value** |
| **Subtype 1** | Yes  No | 4  3 | 26.7  64.4 | 0.222 | 1.10 | 0.38-3.17 | 0.866 |
| **Subtype 2** | Yes  No | 4  2 | 40.2  0.9 | 0.046 | 1.48 | 0.45-4.93 | 0.521 |
| **Subtype 3** | Yes  No | 40  25 | 50.8  29.5 | 0.006 | 1.84 | 0.75-4.50 | 0.182 |
| **Subtype 4** | Yes  No | 88  33 | 53.6  30.0 | 0.000 | 1.92 | 1.10-3.26 | 0.021 |
| **Subtype 5** | Yes  No | 3  0 | -- | -- | -- | ---- | -- |
| **Unclassified** | Yes  No | 45  20 | 57.6  40.2 | 0.082 | 1.36 | 0.56-3.33 | 0.500 |
